# High frequency of exfoliative toxin genes among *Staphylococcus aureus* isolated from clinical specimens in the north of Iran: Alarm for the health of individuals under risk

**Published:** 2018-06

**Authors:** Mojtaba Mohseni, Fariba Rafiei, Ezzat Allah Ghaemi

**Affiliations:** 1 Department of Microbiology, University of Mazandaran, Babolsar, Iran; 2 Department of Microbiology, Golestan University of Medical Sciences, Gorgan, Iran

**Keywords:** *Staphylococcus aureus*, Exfoliative toxin genes, Scalded skin syndrome

## Abstract

**Background and Objectives:**

Exfoliative toxins (ETs) of *Staphylococcus aureus* are the main reason of scalded skin syndrome in infants and young children. The aim of this study was to investigate the prevalence of *eta, etb* and *etd* genes in *S. aureus.*

**Materials and Methods:**

A total of 150 *S. aureus* isolates were collected from clinical specimens during the years 2014 to 2016 in the north of Iran. After confirmation of the species using standard diagnostic procedures, polymerase chain reaction was used for detection of the *eta, etb* and *etd* genes among the isolates.

**Results:**

Overall, 131 (87.3%) isolates were positive for at least one of the ET genes; 115 (76.7%), 25 (16.7%) and 81 (54%) of the isolates carried the *eta, etb* and *etd* genes, respectively. Although *eta* and *etd* genes were present in all types of clinical samples, *etb* was found only in the wound, synovial fluid, sputum and tracheal aspirate. Overall, 7 toxin genotypes were observed, among which the genotypes *eta-etd, eta* and *eta-etb-etd* predominated at rates of 35.3%, 26.7% and 9.3%, respectively.

**Conclusion:**

Detection of the high rate of prevalence of ET genes in the current study is considered as a serious problem because it is likely to spread and transfer these genes between strains. Furthermore, these isolates circulating in the community, particularly from infants, old people and immunocompromised patients, are important health-wise.

## INTRODUCTION

The skin is an essential organ of the human body which has several functions. One of the major functions of the skin is to protect the body against invading pathogens. Furthermore, the skin helps our body to retain necessary fluids and moisture. The cornified layer with the strong adhesion among its keratinocytes cells acts as a barrier against intrusion (1). Removing this protective feature of the skin may cause excessive fluid loss, hypothermia and secondary infection.

Staphylococcal exfoliative toxins (ETs) cause the loss of cohesion between adjacent keratinocytes in the superficial epidermis and to a less extent in the mucous membranes. Damaging the protective epidermis allows the microorganisms to pervade and invade deeper tissues. Such damages can vary from little localized blisters to generalized exfoliation affecting the surface of the body which depends on the presence or absence of protective antitoxins (2). ETs have three isoforms, i.e., ETA, ETB and ETD which are produced by *Staphylococcus aureus* isolated from humans. The *eta* gene encoding ETA has a prophage origin which is located on a chromosome, and the *etd* gene is chromosomally located in a 14.8 kb pathogenicity island. However, the *etb* gene encoding ETB is located on a 42 kb plasmid (3–5). ETs are glutamate-specific serine proteases that specifically cleave desmoglein 1 (Dsg1), a desmosomal intercellular adhesion molecule (6).

From the three isoforms of the ETs, ETA and ETB are the major causative agents of Staphylococcal scalded skin syndrome (SSSS). However, ETD-producing strains of *S. aureus* were isolated mainly from other skin and soft-tissue infections, which are rarely caused by *S. aureus* strains harbouring *eta* or *etb* genes. One can conjecture that ETs play a widespread pathogenic role not only in SSSS, but also in a broad spectrum of cutaneous infections (7). SSSS has two forms; localized and generalised. In the localised form, *S. aureus* produces the toxin locally, whereas in the generalised form, *S. aureus* usually resides in a distal colonised site or in an infective site. The toxins are then released into the bloodstream and a lack of protective antibodies and poor renal clearance allows the toxins to attain the epidermis. Once this is done, they act locally to produce the skin lesions (8).

About 5% of *S. aureus* strains produce exfoliative toxins. Strains producing ETA only, strains producing both ETA and ETB and strains producing ETB only represent 88%, 8% and 4% of toxigenic *S. aureus*, respectively (4, 9). In Europe, Africa and North America, ETA is more widespread; accounting for more than 80% of exfoliative toxin-producing strains, whilst in Japan ETB is more prevalent (2).

PCR procedure is quick, specific and inexpensive to identify the genotype strains harbouring genes for ETA, ETB and ETD. This procedure shows greater sensitivity than various other methods of identifying bacterial toxins such as latex agglutination, latex immunoassay and immunochromatography. The latter methods investigate the production of toxins. In certain conditions, although the bacteria may carry toxin producing genes, it does not have the ability to express it. Hence, the results obtained in the above-mentioned methods will be negative. Therefore, using molecular methods, we can identify strains that produce toxins at low rates (10, 11).

The aims of this study were to investigate the prevalence of *eta, etb* and *etd* genes in *S. aureus* isolated from clinical specimens by PCR molecular analyses.

## MATERIALS AND METHODS

### Bacterial isolates.

A total of 150 *S. aureus* isolates were collected from clinical samples including wounds, blood, sputum, urine, tracheal aspirate, synovial, catheter and ascites samples, from 2014 to 2016 from referral teaching hospitals in Sari and Babol, Iran. The isolates were identified as *S. aureus* using colonial morphology, Gram reaction and catalase, coagulase, DNase and mannitol fermentation tests.

### DNA extraction from culture samples.

Genomic DNA was extracted using the standard bead beating method (12). The cell pellet was mixed with 0.6 ml of CTAB buffer (cetyl trimethyl-ammonium bromide). Then, a 0.5 ml mixture of phenol: chloroform: iso-amyl alcohol (25:24:1) (pH 8.0) was added and transferred into a 2 ml screw cap tube containing 0.5–1.0 g zirconia/silica beads (0.1 mm diameter, Biospec Products Inc.). The tube was beaten using a Mini bead beater (Biospec Products Inc.) at maximum speed for 40 seconds. The top aqueous layer was transferred into a fresh 1.5 ml eppendorf tube after centrifugation at 12000 × g for 5 minutes. Two volumes of polyethylene glycol 6000 (PEG) (30% PEG in 1.5 M NaCl) were added to the tube and left overnight at room temperature. The pellet was collected by centrifugation at 12000 × g for 5 minutes and washed using absolute ethanol. Finally, the pellet containing the nucleic acid was air dried and suspended in 40 μl of ultra pure PCR grade water (Sigma).

### Detection of ET genes using polymerase chain reaction.

The presence of exfoliative toxin A, B and D encoding genes, which are *eta, etb* and *etd* respectively, was detected by PCR using specific primers (Table 1). PCR amplification was undertaken using an MJ Mini thermal cycler (Bio-Rad). The PCR reaction contained 2 μl of DNA template (20 ng μl ^−1^), 2 μl of each primers (10 pM μl^−1^) and 25 μl of master mix (50 units ml^−1^ Taq polymerase, 400 mM dATP, dGTP, dCTP, dTTP and 3 mM MgCl_2_, Promega) then the volume was made up to 50 μl using ultra pure PCR grade water (Sigma). The optimized PCR reactions were performed as follows: 95°C for 5 minutes; followed by 35 cycles of 95°C for 1 minute; 54, 49.9 and 47°C for 1 minute for the *eta, etb* and *etd* genes, respectively; 72°C for 1 minute with a final extension at 72°C for 5 minutes. PCR products were examined using 1% agarose gel stained with ethidium bromide (10 mg ml ^−1^). Electrophoresis was performed at 90 V for 45 minutes. The products were subsequently visualized using a gel documentation system. The 50 bp DNA ladder was used as a DNA size marker. The sizes of PCR products for the *eta, etb* and *etd* fragments were 464 bp, 226 bp and 376 bp, respectively. Positive control strains of ET genes were kindly donated by Dr Imani, Baqiyatallah University of Medical Sciences (13).

**Table 1.** Details of ET gene primers used in PCR experiments.

**Primer^a^**	**Sequence (5′→3′)**	**Product size**	**Reference**
*eta*-F	TTTGCTTTCTTGATTTGGATTC	464 bp	(13)
*eta*-R	GATGTGTTCGGTTTGATTGAC		
*etb*-F	ACAAGCAAAAGAATACAGCG	226 bp	(19)
*etb*-R	GTTTTTGGCTGCTTCTCTTG		
*etd*-F	AACTATCATGTATCAAGG	376 bp	(31)
*etd*-R	CAGAATTTCCCGACTCAG		

a F, forward primer; R, reverse primer.

### Statistical analyses.

Statistical analyses were performed via the statistical package for social sciences (SPSS) software, version 20. The Chi-square test and the Fischer’s test for a lower number series were used to determine the relationship between gender, age and the type of specimen in the presence of the *eta, etb* and *etd* genes. Nominal regression was also used for investigating the differences between coexistence of toxin genes and gender, age and type of the specimen. The significant level was considered to be at p>0.05.

## RESULTS

**Fig. 1.**
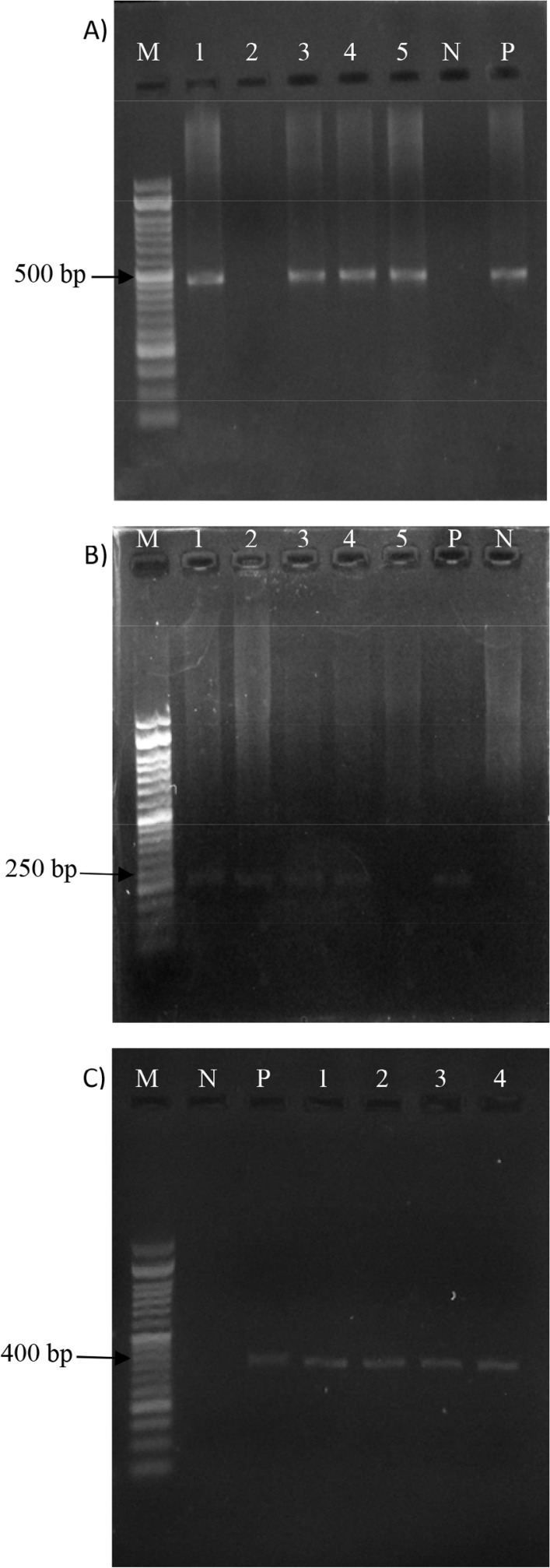
Agarose gel electrophoresis of PCR amplified of the *eta* (A), *etb* (B) and *etd* (C) fragments in *S. aureus* with sizes of 464 bp, 226 bp and 376 bp, respectively. Lane M, 50 bp DNA ladder; lane P, positive control; lane N, negative control; lanes 1–5, some clinical isolates of *S. aureus*.

In this research, the prevalence of Staphylococcal exfoliative toxin genes (*eta, etb* and *etd*) in the clinical isolates of *S. aureus* was investigated using PCR method. The presence and occurrence of the ampl-icons using agarose gel electrophoresis is shown in Fig. 1. The sizes of the PCR products were equal to those predicted from the designed primers (Table 1).

**Fig. 2.**
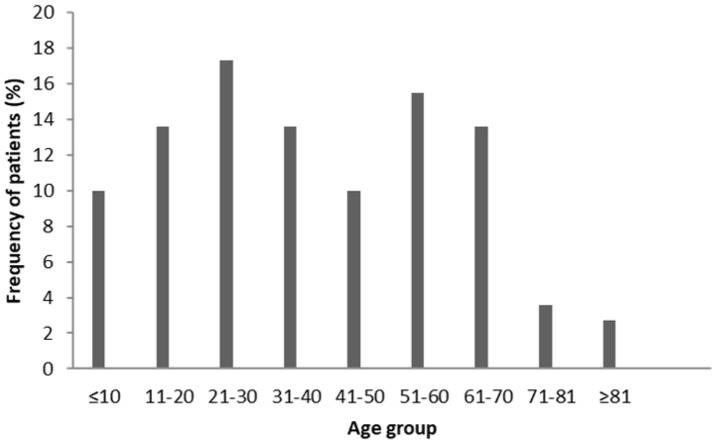
The frequency of the patients with respect to age range.

**Fig. 3.**
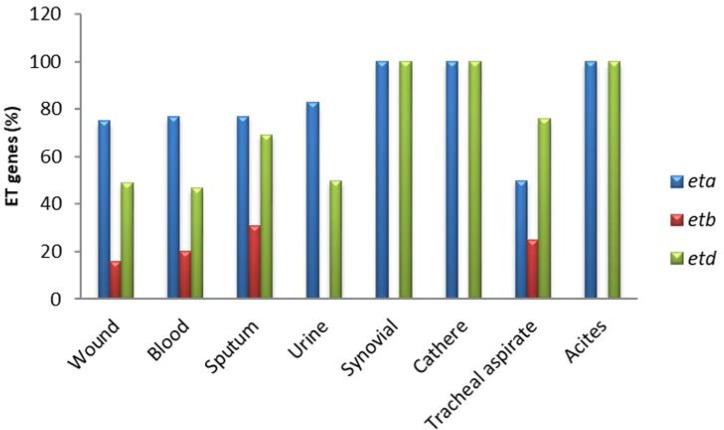
The frequency of ET genes in isolated *S. aureus* based on different specimens.

Of 150 *S. aureus* which were obtained from different patients; 88 (58.6%), 30 (20.0%), 13 (8.6%), 6 (4.0%), 4 (2.6%), 4 (2.6%), 4 (2.6%) and 1 (0.6%) isolates were taken from wounds, blood, sputum, urine, tracheal aspirate, synovial, catheter and ascites samples, respectively. Furthermore, 32% of patients were female and 68% were male. The frequency of the patients with respect to age range is shown in Fig. 2. with the most frequent belonging to the age group of 21–30 years. Among the exfoliative toxin genes, the *eta* gene (n: 115, 76.7%) was the most widespread, followed by the *etd* (n: 81, 54.0%), and *etb* genes (n: 25, 16.7%). Of *eta*-carrying strains, 79.6% were obtained from males and 69.6% were obtained from females. In addition, the *etb* and *etd* genes were detected in 16.3% and 51% of males and 17.4% and 58.7% of females, respectively. A higher number of *eta*-positive strains were isolated from male samples and a higher prevalence of *etb* and *etd* positive strains were found in the females samples. In all isolates obtained from wound, blood, sputum and urine specimens, *eta* gene is more frequent than other *et* genes. However, in the isolates obtained from tracheal aspirate specimens, the frequency of *etd* gene is more than other *et* genes (Fig. 3). Furthermore, the frequency of *eta* and *etd* genes is identical in isolates from the synovial, catheter and ascites samples, yet none of them carries the *etb* gene. Therefore, it is concluded that prevalence of the *etb* gene in *S. aureus* isolated from sputum (30.7%), tracheal aspirate (25.0%) and blood (16.7%) samples is higher than isolates obtained from other samples. About 80% of strains isolated from blood samples carried at least one of the *et* genes that have the least amount of carrier compared to isolates obtained from other clinical specimens. In other words, more than 80% of strains isolated from wounds, urine, sputum, synovial, catheters, tracheal aspirate and ascites samples were carriers of at least one of the *et* genes. However, there were no significant differences between gender, age and the type of specimen in the presence of the *eta, etb* and *etd* genes.

The prevalence of toxin gene coexistence in the strains isolated from different clinical specimens is shown in Table 2. Overall, 7 toxin genotypes were observed, among which the genotypes *eta, etd, etaetd* and *eta-etb-etd* predominated at rates of 26.7%, 8.7%, 35.3% and 9.3%, respectively. Of the 150 isolates, 19 (12.7%) lacked *et* genes, while 14 (9.3%) expressed all three *eta, etb* and *etd* genes. The combination of *eta-etd* (35.3%) genes is more common among isolates than *eta-etb* (5.3%) or *etb-etd* (0.7%) genes. More than half of these *eta*-positive isolates (58.3%) were carriers of the *etd* gene. Furthermore, 82.7% of *etd*-positive isolates were carriers of the *eta* gene. About 88% and 60% of *etb*-positive isolates also carried *eta* and *etd* genes, respectively. Among combinations of ET genes, genotype *eta* predominated in isolates obtained from wound and urine samples, while genotype *etd* predominated in isolates obtained from tracheal aspirate samples. Furthermore, the most common genotype among isolates obtained from other clinical samples was *eta-etd.* All of the isolates from the synovial, catheter and ascites samples indicated only genotype *eta-etd.* A combination *etb-etd* gene was detected only in one isolate, which was obtained from blood. In addition, genotype *etb* was detected only in isolates obtained from wound samples. The genotype *eta* in the strains isolated from males was found to be about four times higher than females (*p*=0.035, odds ratio=3.771). No other significant differences were observed between toxin gene combinations, gender, age and type of specimen.

**Table 2.** Distribution of toxin genes in *S. aureus* from different human clinical specimens.

	**No. of *S. aureus* isolates**																	

**Gene combination**	**Wound (n: 88)**		**Blood (n: 30)**		**Sputum (n: 13)**		**Urine (n: 6)**		**Snovial (n: 4)**		**Catheter (n: 4)**		**Tracheal aspirate (n: 4)**		**Ascites (n: 1)**		**Total (n= 150)**	

	**n**	**(%)**	**n**	**(%)**	**n**	**(%)**	**n**	**(%)**	**n**	**(%)**	**n**	**(%)**	**n**	**(%)**	**n**	**(%)**	**n**	**(%)**
*eta*	29	(32.9)	7	(23.3)	1	(7.7)	3	(50)	0	(0)	0	(0)	0	(0)	0	(0)	40	(26.7)
*etb*	2	(2.3)	0	(0)	0	(0)	0	(0)	0	(0)	0	(0)	0	(0)	0	(0)	2	(1.3)
*etd*	9	(10.2)	0	(0)	1	(7.7)	1	(16.7)	0	(0)	0	(0)	2	(50)	0	(0)	13	(8.7)
*eta, etb*	3	(3.4)	3	(10)	1	(7.7)	0	(0)	0	(0)	0	(0)	1	(25)	0	(0)	8	(5.3)
*eta, etd*	25	(28.4)	11	(36.7)	5	(38.5)	2	(33.3)	4	(100)	4	(100)	1	(25)	1	(100)	53	(35.3)
*etb, etd*	0	(0)	1	(3.3)	0	(0)	0	(0)	0	(0)	0	(0)	0	(0)	0	(0)	1	(0.7)
*eta, etb, etd*	9	(10.2)	2	(6.7)	3	(23.1)	0	(0)	0	(0)	0	(0)	0	(0)	0	(0)	14	(9.3)
Total positive	77	(87.5)	24	(80)	11	(84.6)	6	(100)	4	(100)	4	(100)	4	(100)	1	(100)	131	(87.3)
Total negative	11	(12.5)	6	(20)	2	(15.4)	0	(0)	0	(0)	0	(0)	0	(0)	0	(0)	19	(12.7)

## DISCUSSION

*S. aureus* is known as the main nosocomial pathogen due to the production of a vast range of virulence factors such as extracellular protein toxins. Furthermore, *S. aureus* causes significant mortality and morbidity due to severe nosocomial infections (14). *S. aureus* produces many factors such as ETs that contribute to the spread of bacteria, host colonization and tissue attack (15). In the current study, ET genes both alone and in various combinations were detected in clinical *S. aureus* isolates by using the PCR method. A majority of the isolates (58.7%) were collected from wound samples. The genotyping outcomes demonstrated an exceedingly high outbreak of *eta, etb* and *etd* genes in the *S. aureus* isolated from clinical specimens (76.7%, 16.7% and 54.0%, respectively). This prevalence rate is higher than those found by Becker et al. in Germany, Liu et al. in China, Kolawole et al. in Nigeria and Mehrotra et al. in Canada (16–19). In the mentioned studies, *eta* and *etb* genes were commonly observed at ranges of 1.2%–4.0% and 0.5%–1.7%, respectively. Of course, no *etd* gene was detected in Nigeria. It is noteworthy that in all these cases, the number of wound samples was near zero and they used different kinds of samples to conduct their research. Wu et al. (2011) in China and van Trijp et al. (2010) in the Netherlands found the *eta* gene in 1% and 2% of the isolates, respectively. Van Trijp et al. also found the *etd* gene in 4.5% of strains, but Wu et al. did not find any *etd* gene in the strains (20, 21). In these studies, the highest number of isolates was collected from the respiratory tract. The prevalence of *eta* and *etd* genes in *S. aureus* obtained in the above studies is fewer than the *eta* (76.7%) and *etd* (54.0%) genes in the *S. aureus* observed in our study. In Switzerland, the low rates of positive isolates containing *eta* (2%), *etb* (2%) and *etd* (5%) were detected among 101 methicillin-sensitive *S. aureus* (MSSA), which were isolated from infected patients (22).

In another study in the United Kingdom, Peacock et al. investigated the prevalence of the *eta* and *etb* genes among isolates recovered from 155 patients with invasive *S. aureus* disease. In this study, 22% of the isolates carried the *eta* gene, but no strain carried the *etb* gene. They also investigated the prevalence of these genes among isolates recovered from 179 healthy individuals; only 6% and 3% of the isolates carried *eta* and *etb* genes, respectively (23). Therefore, the relatively low prevalence of genes has been reported in different countries. However, higher prevalence of *eta* and *etb* genes among isolates has been reported in Turkey (24). They observed a prevalence of 19.2% and 9.2% for *eta* and *etb* genes, respectively. Unlike previous studies, in the above study, the majority of isolates were collected from wound samples, similar to our study. High prevalence of ET genes may be explained by the fact that Staphylococcal exfoliative toxins are specific serine proteases that act as “molecular scissors” to facilitate colonization and bacterial invasion through the mammalian skin and mucosa by cleavage of adhesion molecules between adjacent keratinocytes (1). Thus, the high rates of exfoliative toxin genes in the current study could be due to the high frequency of wound specimens (13). Therefore, a large number of carrier isolates from wound specimens indicates a secondary role for *S. aureus* in the development of these wounds.

In a study conducted by Yamasaki et al. in France, the *etd* gene was found in 55 (10.5%) of the 522 *S. aureus* isolates. About 48 of the 445 isolates taken from patients, and 7 of the 77 colonization isolates, were *etd* carriers. Most these carriers were isolated from skin infections that suggest the role of this gene in the development of these infections (7). The frequency of the *etd* gene in our isolates was 54%. These differences could also be due to geographical differences. Although these genes can exist in different genotypes, their prevalence is higher in some clones and gene clusters. Furthermore, geographical distribution and transmission of these clones in distinct regions are different. Some strains are dominant in certain areas and some of them have global outbreaks (18, 20, 25).

In Iran, several studies have been conducted in this respect in recent years. The frequency rate of the *eta* gene in studies performed in Shiraz, Ilam, Shahrekord and Arak was 0.68%, 1.0%, 7.6% and 14.3%, respectively. In addition, the results for the *etb* gene were 2.05%, 15.3% and 3%, respectively, while no isolate carries the *etb* gene in Arak (26–29). In all cases, the *eta* and *etb* gene frequency was lower than our results (77.3% and 16.6%, respectively). However, our findings for the *etb* gene frequency are almost identical to the results of a survey conducted in Shahrekord. Most samples examined in Shiraz and Shahrecord, are collected from sputum and nasal swab, respectively.

Similar results were reported in Baqiyatallah hospital in the north of Tehran by Koosha et al., 2014. Out of 197 isolates, 186 (94.4%) and 15 (7.6%) isolates harboured *eta* and *etb* genes, respectively. In the stated study, the majority of the isolates were taken from wound samples; thus, 85 (43.1%) isolates were obtained from wound samples (13). The prevalence rate of the *eta* gene is higher than our results. However, the frequency rates of *eta* and *etb* genes among 133 strains isolated from different parts of an Iranian referral children’s hospital in Tehran were 11.3% and 9%, respectively (30). In the current study, the frequency of the *eta* and *etb* genes was 76.7% and 16.7%, respectively.

The results of the research conducted at the centre, west and south of Iran indicated the low frequency of ET genes in these areas. While the high prevalence of these genes was observed in Tehran and Mazandaran which are both located in the north of Iran. Since ET-encoding genes are carried on prophage, plasmids and pathogenicity islands as mobile genetic elements (MGEs), which enable transfer into *S. aureus* lineages via horizontal transfer, the north of Iran may act as a potential major reservoir for the ET virulence factor. This is another reason for the high prevalence of these genes (14).

In addition, *S. aureus* isolated from nose and throat swabs of several nurses in the hospital were evaluated; the results showed that more than half of these isolates were positive for these genes. This can be the reason for such a high frequency rate of these genes within the isolates. According to the current study, as well as studies in other Iranian cities, it is concluded that *eta*, compared to *etb* and *etd*, is more widespread in Iran.

Although ET genes are highly prevalent among the isolates, the lack of SSSS among our population suggests that the adequate amount of toxins required to cause SSSS is not being produced. For example, it was shown that *S. aureus* isolates from the nose and throat swabs of nurses harboured high ratio of toxin genes, however, none of them display symptoms of the disease (SSSS). In addition, the presence of the toxin genes does not show any expression level for the related protein (24). Also, it is feasible that either a stimulating factor in the host or organism may be needed for toxin production, or the organism may continually be producing the toxin but the toxin is prevented from reaching the epidermis, either locally or through the blood (8).

Due to the high prevalence of these genes in the present study, the investigation of SSSS outbreak in the region is suggested.

In conclusion, our results indicated that ET genes were very common among *S. aureus* isolates collected from clinical specimens. The high outbreak of ET genes in clinical specimens in Iran is considered a serious problem. Because all of the ET genes were encoded by certain MGEs, it is likely that they spread and transfer these genes between strains. These isolates that are circulating in the community, particularly the adults who have serious underlying medical problems such as immunosuppression or renal dys-function, are important health-wise. This subject becomes more significant considering the high rate of colonization of this bacterium in healthy individuals. The examination of these isolates in hospitals can be effective for people under risk control.
